# *Candida* species isolated from clinical samples in a tertiary hospital in Honduras: Where is *Candida auris*?

**DOI:** 10.18502/cmm.8.3.11212

**Published:** 2022-09

**Authors:** Bryan Ortiz, Kateryn Aguilar, Celeste Galindo, Lizzy Molina, Gustavo Fontecha

**Affiliations:** 1 Microbiology Research Institute, Universidad Nacional Autónoma de Honduras, Tegucigalpa, Honduras; 2 Instituto Hondureño de Seguridad Social, Tegucigalpa, FM, Honduras; 3 Escuela de Microbiología, Universidad Nacional Autónoma de Honduras, Tegucigalpa, Honduras

**Keywords:** Cryptic species, *Candida albicans* complex, hwp1 gene, *gpi* gene

## Abstract

**Background and Purpose:**

Infections by emerging and multiresistant *Candida* species are becoming more frequent throughout the world. This study aimed to describe *Candida* species in different wards of a tertiary hospital in Honduras.

**Materials and Methods:**

The prevalence of species within the *C. albicans* complex was estimated using a molecular approach, and *C. auris* was investigated using a yeast pool-based DNA extraction method. In total, 328 yeast isolates were identified using phenotypic approaches. For the identification of species within the *C. albicans* complex, a molecular approach based on the size polymorphisms of the hpw1 gene was used. In addition, a technique was optimized based on DNA extraction in pools for the rapid identification of *C. auris*.

**Results:**

A total of 11 species of *Candida* were identified in the hospital wards. *C. albicans* showed the highest number of isolates (52.4%). Within the *C. albicans* complex, *C. albicans* sensu stricto was the most common, followed by *C. dubliniensis*. However, *C. auris* was not found.

**Conclusion:**

Reports on the distribution of *Candida* species in Honduras are limited; accordingly, the data from this study are of importance for a better understanding of their epidemiology. Moreover, a simple method was offered for the detection of *C. auris* that could help in its detection in low-resource settings.

## Introduction

The incidence of fungal infections has increased significantly in recent decades, particularly those caused by Candida species [ 1 , 2 ]. Candidiasis range from mild superficial to life-threatening invasive infections [ 3 ], with an annual prevalence of tens of millions of cases and mortality rates above 40% [ 4 , 5 ]. More than 300 species belong to the genus *Candida* [ 6 ]. In the 1990s, human *Candida* infections were limited to five species, namely *C. albicans*, *C. glabrata*, *C. parapsilosis*, *C. tropicalis*, and *C. krusei* [ 7 , 8 ]; however, thanks to advances in molecular diagnostic tools, more than 37 species have been reported [ 9 ]. 

*C. albicans* remains the most frequently isolated species [ 10 , 11 ]; however, the epidemiology of *Candida* seems to have undergone recent changes [ 12 ]. Infections caused by species other than *C. albicans* have increased markedly, accounting for more than 50% of isolates recovered from blood cultures [ 11 ]. This increase could be due to the widespread use of broad-spectrum antibiotics, immunosuppressive agents, prolonged hospitalization, chemotherapy, and more recently, patients infected with SARS-CoV-2 [ 4 , 11 , 13 ]. 

*C. glabrata*, *C. parapsilosis*, *C. tropicalis*, *C. krusei*, *C. guilliermondii*, and *C. lusitaniae* are the most common causes of infections caused by non-*albicans* species [ 9 , 11 , 14 ]. *C. auris*, a multi-resistant pathogen described in 2009, has been added to this list of non-*albicans* yeasts that are most frequently isolated in the hospital environment [ 9 , 13 , 14 ] due to its high capacity to indefinitely colonize various anatomical locations, which facilitates its transmission between patients and the appearance of long-term outbreaks. On the other hand, it is estimated that around 25% of patients colonized by *C. auris* may develop candidemia and/or invasive candidiasis. In addition, the epidemiological relevance of this species is due to the high resistance to antifungals and disinfectants and the difficulties to identify it by the phenotypic methods available in routine laboratories [ 15 , 16 ]. This is a worrying scenario for public health, mainly due to the difference in the sensitivity profiles of these species against the available therapies [ 17 ], making it difficult to manage patients, resulting in high mortality rates and high financial costs [ 2 , 18 ]. 

Sustained surveillance and monitoring of the epidemiology of clinically important yeasts should be a priority, particularly in countries considered low- and middle-income where yeast identification methods are limited. Knowledge of the local epidemiology of *Candida* species is essential for adequate prevention and clinical management since in many cases the identification of species allows predicting their potential susceptibility to antifungals.

The main objective of this study was to describe, through a phenotypic approach, the *Candida* species circulating in the wards of a hospital in Honduras. In addition, the prevalence of species within the *C. albicans* complex was estimated, and the presence of *C. auris* was investigated using molecular methods and a DNA extraction methodology based on yeast pools.

## Materials and Methods

### Clinical samples

A total of 328 axenic yeast cultures from a tertiary hospital (Instituto Hondureño de Seguridad Social) were evaluated between May 2021 and March 2022. Cultures showing predominant yeast presence were included. Yeasts cultured from clinical samples included urine (n=146), sputum (n=78), blood (n=38), feces/rectal exudates (n=16), vaginal discharge (n=14), tracheal aspirates (n=4), bronchial secretion (n=3), catheters (n= 2), oral swabs (n= 2), abdominal discharge (n= 2), ulcer discharge (n=2), surgical wound discharge (n=2), ascitic fluid (n=2), peritoneal fluid (n=1), foot discharge (n=1), tongue discharge (n=1), urethral discharge (n=1), and other secretions (n=13). 

Regarding ethical considerations, the study was conducted on anonymous biological samples. Furthermore, this research did not involve any personal data that could directly or indirectly identify a specific individual. Therefore, a statement of consent from the ethics committee of the hospital or university was not necessary. Clinical samples were collected according to ethical standards and due to the retrospective nature of the study, the consent form was not applicable to the study.

### Yeast Identification

Clinical samples were cultured on blood agar, MacConkey agar, and chocolate agar. When yeasts were observed on Gram stain or wet smear, both in cultures or in clinical samples, the samples were plated on CHROMagar^TM^ *Candida* Medium (Becton, Dickinson and Company, NJ, USA). Blood cultures were carried out in BACTEC flasks Peds Plus/F Culture Vials (Becton, Dickinson and Company, NJ, USA). Blood cultures were monitored by the automated system BD BACTEC^TM^ FX (Becton, Dickinson and Company, NJ, USA) for five days. Cultures were incubated from 24 to 48 h at 37°C under aerobic conditions.Yeast species were preliminarily identified by CHROMagarTM Candida chromogenic medium (Becton, Dickinson and Company, NJ, USA), which identifies the main species of *Candida* by the color of the colonies. CHROMagar^TM^ (Becton, Dickinson and Company, NJ, USA) cultures were incubated at 37°C for 48 h and scored for colony color according to the manufacturer's instructions. For the definitive identification of the yeasts, the BD Phoenix yeast identification system BD PhoenixTM (Becton, Dickinson and Company, NJ, USA) was used. This system is capable of identifying up to 64 species.

With the isolated colonies, a suspension was made with a density equivalent to the McFarland standard Nº2. Suspensions were then inoculated onto yeast identification panels and loaded onto the BD Phoenix^TM^ M50 instrument (Becton, Dickinson and Company, NJ, USA). Results were registered after 4 to 8 h. The prevalence of *Candida* species was determined according to the hospital ward and gender of patients.

### Detection of Candida species from yeast pools

To assess the presence of three species of the *C. albicans* complex (*C. albicans* sensu stricto, *C. dubliniensis*, and *C. africana*), the approach proposed by Theill et al. was used [ 19 ] with slight modifications. This method is based on the combination (pooling) of individual axenic cultures for the detection of a target species. This approach was also used to detect C. auris.

**Figure 1:**
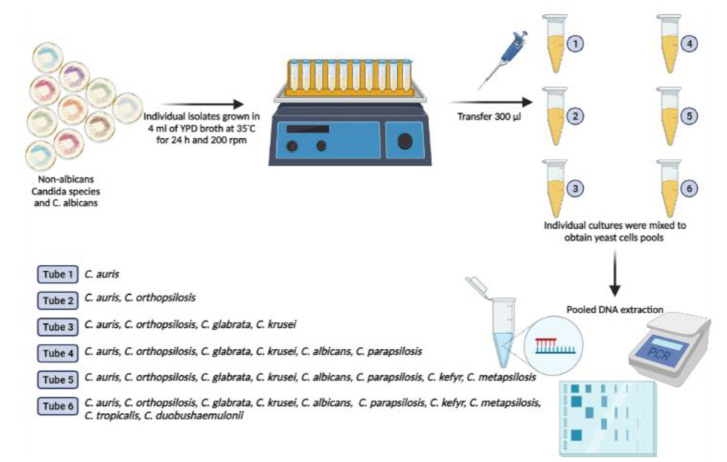
Protocol used to demonstrate the ability of the yeast pool method to detect *Candida auris*. Created with Biorender.com

To optimize the pooling method and demonstrate its sensitivity in detecting *C. auris*, 10 *Candida* species, including a reference strain of *C. auris*, were cultured individually in YPD liquid medium (1% yeast extract, 2% peptone, and 2% dextrose) at 30°C for 24 h under constant stirring at 200 rpm. Individual cultures were arranged in pools that included 300 µL of each culture. Six different pools were tested (Figure 1). Once the pools were prepared, DNA extraction was carried out using a previously described approach based on phenol chloroform [ 20 ].

Molecular identification of species within the albicans complex

Isolates identified as *C. albicans* were grown individually in 4 mL YPD broth at 30°C for 24 h with constant shaking at 200 rpm. 300 µL of each culture were taken and mixed in pools of 10. DNA was extracted from each pool according to a previously published protocol [ 20 ]. 

The identification of the species within the *albicans* complex was based on a size polymorphism in the hyphal wall protein 1 gene (hwp1) [ 21 ]. DNA was amplified in a volume of 50 µL, with 25 µL of 2× PCR

Master Mix (Promega Corp. Madison, WI, USA), 19 µL of nuclease-free water, 2 µL of DNA (40 ng/µL), and 2 µL of each primer 10 µM: CR-f: 5′-GCTACCACTTCAGAATCATCATC-3′ and CR-r: 5′-GCACCTTCAGTCGTAGAGACG-3′. PCR conditions were: denaturation at 95°C for 5 min, 35 cycles at 94°C for 45 s, 58°C for 40 s, and 72°C for 55 s, with a final extension at 72°C for 10 min. PCR products were separated and visualized on an ethidium bromide agarose gel. PCR products with a size of 941 bp were identified as *C. albicans sensu stricto*, while products of 569 bp and 750 bp were identified as *C. dubliniensis* or *C. africana*, respectively. Additionally, individual cultures were stored at -20ºC, and when the pools showed a different identification to *C. albicans sensu stricto*, DNA extraction was performed individually from the strains included in that pool in order to confirm the species of each isolate.

### Molecular detection of C. auris

Briefly, pools were prepared using 10 yeasts previously identified as non-*albicans* species or as *C. tropicalis* by the BD Phoenix Yeast Identification System or CHROMagar^TM^ *Candida* Medium (Becton, Dickinson and Company, NJ, USA). This decision was based on previous reports of white to mauve colonies of *C. auris* on CHROMagar^TM^ (Becton, Dickinson and Company, NJ, USA), and because identification has not yet been optimized for any clade of *C. auris* in the BD Phoenix Yeast Identification System (Becton, Dickinson and Company, NJ, USA), this yeast could be misidentified as *C. catenulata*, *C. haemulonii*, or *C. parapsilosis* [ 15 , 22 , 23 ]. 

The identification of *C. auris* was carried out by amplifying the gene glycosylphosphatidylinositol (*gpi*) [ 24 ]. A mix was prepared with 12.5 µL of 2× Master Mix (Promega Corp. Madison, WI, USA), 9 µL of nuclease-free water, 1.5 µL of DNA (40 ng/µL), and 1 µL of each primer 10 µM: 03410_F: 5′-GCCGCTAGATTGATCACCGT-3′ and 03410_R:5′-TAGGTGTGGGTACCCTTGGT-3′. The PCR program consisted of 1 cycle at 94°C for 3 min, 35 cycles at 94°C for 30 s, 60°C for 30 s, 72°C for 30 s, and a cycle at 72°C for 3 min. A band size of 137 bp was indicative of *C. auris*, and no amplification product was expected for the rest of the species. Positive and negative controls were included in each PCR reaction.

## Results

### Distribution of Candida species

In total, 11 *Candida* species were identified. *C. albicans* was the most frequent (n=172; 52.5%) and non-*albicans* species added the remaining 47.5%. *C. tropicalis* (13.4%), *C. krusei* (11.2%), and *C. glabrata* (11%) were the most frequent non-*albicans* species. *C. pararugosa*, *C. guillermondii*, and *C. fimetaria* showed the lowest number (Table 1). Isolates of non-*albicans* species were more frequent in women (66.4%) than in men (33.6%).

**Table 1:** Distribution of *Candida* species isolated between 2021 and 2022 in the IHSS hospital

Yeast species	n (%)
*C. albicans*	172 (52.5)
*C. tropicalis*	44 (13.4)
*C. krusei*	37 (11.2)
*C. glabrata*	36 (11)
*C. parapsilosis*	19 (5.8)
*C. lusitaniae*	10 (3)
*C. dubliniensis*	5 (1.5)
*C. pelliculosa*	2 (0.6)
*C. pararugosa*	1 (0.3)
*C. guilliermondii*	1 (0.3)
*C. fimetaria*	1 (0.3)

**Figure 2:**
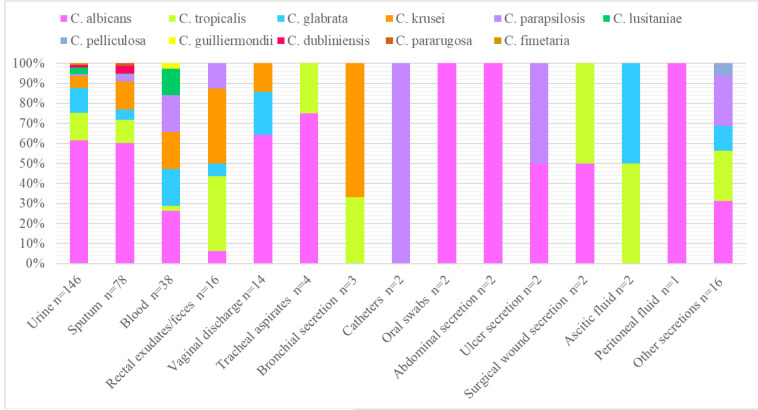
Distribution of *Candida* species by clinical samples

Yeasts were most frequently isolated from urine (44.5%), sputum (23.7%), and blood (11.5%). *C. albicans* was the most frequent species in most of the samples with the exception of rectal exudates/feces and blood, in which the non-*albicans* species were more prevalent. Sputum and urine were the samples with the greatest diversity of species (Figure 2).

Since 80% of the isolates were from urine, sputum, or blood, the distribution of *Candida* species in these samples was analyzed according to the hospital ward. The wards with the highest number of isolates in urine were internal medicine (26%), emergency (21%), and gynecology (14.3%). 

The most frequent non-*albicans* species in urine were *C. tropicalis*, *C. glabrata*, and *C. lusitaniae*. The internal medicine (44%), emergency (30%), and COVID-19 (10.3%) wards contributed more isolates from sputum. The non-*albicans* species most frequently isolated from sputum were *C. krusei*, *C. tropicalis*, and *C. glabrata*.

Internal medicine (29%), emergency (21%), and pediatrics (18.4%) had the highest number of isolates from blood cultures. Non-*albicans* species (*C. glabrata*, *C. krusei*, and *C. parapsilosis*) accounted for 73.6% of bloodstream infections. Two cases of mixed candidemia were found, one caused by *C. krusei* / *C. lusitaniae* in a patient in the COVID-19 ward, and another case caused by *C. glabrata* / *C. parapsilosis* from pediatrics. Cases of mixed candidemia accounted for 5.3% of isolates from blood cultures. The prevalence of candidemia in the ICU was 10.5%.

### Identification of C. albicans complex and C. auris

Overall, 177 (54%) isolates were identified as *C. albicans* complex, of which 172 were C. albicans sensu stricto (97.2%). Five (2.8%) isolates were identified as *C. dubliniensis*. Isolates of *C. dubliniensis* were obtained from sputum (n=3) and urine (n=2). *C. africana* was not found. 

**Figure 3:**
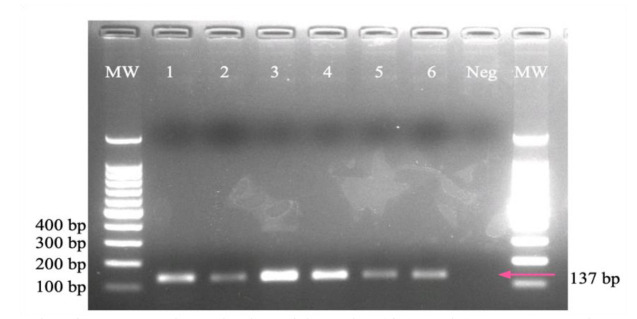
Agarose gel electrophoresis. Lane 1 and 9 molecular weight marker (size 100 bp). Lanes 2 to 6 show a 137 bp band as a result of amplification of the GPI gene from the reference strain of *C. auris* from pooled DNA from up to 10 *Candida* species

Regarding *C. auris*, a simple and inexpensive method based on DNA extraction from mixed cultures was optimized for the detection of suspect isolates. In the six pools prepared to optimize the method, *C. auris* DNA was successfully amplified (Figure 3). A total of 112 yeast isolates suspected of being *C. auris* were evaluated; however, it was not demonstrated in any case.

## Discussion

*C. albicans* was the most frequently isolated species. This result is consistent with a previous study carried out in the same hospital in 2019 [ 25 ], as well as with several studies from other countries [ 3 , 26 - 29 ]. Although the incidence of non-*albicans* species seems to be increasing [ 9 , 11 , 30 ], our results reveal that *C. albicans* remains the most frequently isolated species from clinical samples in Honduras. *C. tropicalis*, *C. krusei*, and *C. glabrata* were the most commonly isolated yeasts after *C. albicans*. The incidence of these species agrees with a previous study carried out in Honduras where 167 cultures were evaluated, with *C. albicans*, *C. tropicalis*, and *C. glabrata* being the most commonly isolated species [ 25 ]. Similar results have been reported in Mexico and India [ 27 , 31 , 32 ]. 

*C. krusei* represented 11.3% of isolates and was the third most frequent species. This represents a two-fold increase in the number of isolates, compared to the 2019 study when 4.5% of isolates were reported from the same hospital [ 25 ]. This difference could be attributed to the two-year difference between both studies, or it could also be influenced by the indiscriminate use of azoles, particularly fluconazole, which has been related to the increase in *C. krusei* due to selective pressure exerted on sensitive strains [ 33 ]. The scope of the present study was not intended to confirm this hypothesis, and future studies would probably delve into the reasons for these changes in the epidemiology of non-*albicans* species.

Urine, sputum, and blood were the samples in which yeasts were isolated most frequently. Our results coincide with two available studies on the epidemiology of *Candida* in Honduras [ 20 , 25 ] in which *C. albicans* is described as the predominant yeast in urine and sputum. Similarly, our results are also similar to those previously reported elsewhere [ 32 , 34 , 35 ]. The clinical significance of yeasts in urine and sputum is still not entirely clear, as it could indicate contamination, commensalism, colonization, or even a localized infection that could later evolve into a disseminated infection [ 20 , 36 ]. Whatever the meaning of isolating yeasts in urine or sputum, it is important to report their presence so that the clinician has a complete picture of the microbiota in the patient. 

Two studies have evaluated species causing candidemia in Honduras. In a laboratory-based survey conducted at 20 centers in seven Latin American countries, nine species of *Candida* were reported to cause candidemia among 135 cases in Honduras [ 37 ], while Montes et al. reported five species among 12 cases [ 25 ]. Here, we identify seven species isolated from the bloodstream. In three studies, the non-*albicans* species were the most frequent in blood, widely surpassing *C. albicans*. These data support those reported by Pappas et al., where non-*albicans* species could cause more than 50% of candidemia cases worldwide [ 11 ]. 

*C. glabrata*, *C. krusei*, and *C. parapsilosis* were found in equal proportion, disputing the second place as causative agents of candidemia. Infections with *C. glabrata*, *C. krusei*, and *C. parapsilosis* have been associated with mortality rates ranging from 10% to 40% [ 33 ]. *C. glabrata* causes 5%-20% of candidemia cases worldwide and is the second leading cause of candidemia in the United States and Northern Europe [ 38 , 39 ]. In addition, this yeast is associated with elderly patients and transplant recipients [ 33 , 40 ]. 

In recent years, the increased prevalence of *C. glabrata* resistant to azoles and echinocandins has been reported [ 37 , 40 - 42 ]. *C. krusei* is commonly associated with infections in patients with hematologic malignancies who are undergoing treatment with fluconazole, echinocandins, or antibacterial agents, specifically vancomycin or piperacillin-tazobactam. It also has intrinsic resistance to fluconazole; and *in vitro* high minimal inhibitory concentrations against amphotericin B have been observed [ 33 , 37 , 43 ].

*C. parapsilosis* is the second most frequently isolated species in Asia, Latin America, and in some hospitals in southern Europe [ 37 , 40 , 41 , 44 ], whereas in North America, it has been considered the third most frequent [ 45 ]. Infections by *C. parapsilosis* predominate in low-birth-weight newborns, critically ill or cancer patients [ 44 , 46 ]. Although in most reports *C. parapsilosis* tend to be sensitive to available antifungals, it has reduced susceptibility to echinocandins [ 47 , 48 ]. Secondary resistance to azoles in *C. parapsilosis*, particularly fluconazole is also becoming frequent [ 49 - 51 ]. Our results highlight the need for a comprehensive effort to assess the distribution of *Candida* species and risk factors associated with systemic candidiasis in Honduras. Antifungal susceptibility profiles need to be routinely assessed as well, as non-*albicans* species could be associated with increased resistance [ 17 ].

Two cases of mixed candidemia were demonstrated. Most cases of candidemia are usually monomicrobial, while cases of mixed candidemia are rare [ 52 , 53 ]. Within the candidemia cases, the estimated prevalence of mixed candidemia is usually less than 10% [ 53 , 54 ], which makes this finding an unusual event.

In this study, the prevalence of the three species of the *C. albicans* complex was determined. Historically, *C. albicans* has been considered the main causal agent of superficial and invasive candidiasis [ 9 , 41 ]; however, isolates identified as *C. albicans* had phenotypic and genetic inconsistencies at the time of their identification, which is why they were called atypical isolates of *C. albicans* [ 55 , 56 ]. Due to advances in molecular diagnostic tools, atypical strains were later considered new species within a complex [ 55 , 56 ]. Hence, the epidemiology of *C. albicans sensu stricto* may have been historically overestimated. Within the isolates classified in the albicans complex, 2.8% were *C. dubliniensis*, while *C. africana* was not found. Fontecha et al. reported 5% of *C. dubliniensis* and 3% of *C. africana* among 66 yeasts isolated in the same hospital [ 57 ]. 

Since *C. dubliniensis* was first identified in Ireland as the cause of oropharyngeal candidiasis in patients living with HIV, it has been identified in many other countries [ 19 , 28 , 57 - 61 ]. The pathogenesis and antifungal susceptibility of *C. dubliniensis* are not clearly defined. Several studies suggest that *C. albicans* and *C. dubliniensis* share the same in vitro antifungal susceptibility patterns, as well as clinical breakpoints and epidemiological Cut-Off values [ 19 , 58 , 62 - 63 ]; however, Ayadi et al. studied the in vitro reaction of *C. albicans* and *C. dubliniensis* against fluconazole and two echinocandins and observed that both yeasts behave differently [ 64 ]. Likewise, the comparative analysis of the genomic and transcriptomic data has revealed important differences in their infective capacity [ 65 ]. 

A technique was optimized based on pools in order to offer an easy and economical method to identify *C. auris*. This method was then applied to screen 112 suspected isolates of *C. auris*, and as reported in 2017 and 2019, it was not possible to demonstrate the presence of *C. auris* in the country yet [ 20 , 57 ]. Consequently, the evidence seems to suggest that *C. auris* is not yet found in Honduras, or at least in the hospital where this study was carried out. However, the report of *C. auris* in other Latin American countries [ 66 ], plus the recent association of outbreaks by *C. auris* in the context of the COVID-19 pandemic [ 66 ], makes its active search a priority for the country. Having tools that allow the timely identification of *C. auris* when the first cases appear will guarantee quick decision-making for its management and control.

The main limitation of this study is that the data were collected from a single hospital in Tegucigalpa; therefore, our results cannot necessarily be extrapolated to the epidemiological situation of candidiasis in the country. Another limitation is the lack of identification of cryptic species of the *C. parapsilosis* and *C. glabrata* complexes.

## Conclusion

This study updated the distribution of *Candida* species isolated from clinical samples and provided data to better understand the epidemiology of *Candida* species in Honduras. In addition, a simple and inexpensive technique was offered for the detection of *C. auris* that could help in the rapid detection of this pathogen in low-resource laboratories. It is suggested that decision-makers remain alert to the possible appearance of *C. auris* in Honduras.

## Acknowledgments

The authors acknowledge the support given by the staff of the IHSS hospital in Tegucigalpa. No funding was received for this study.

## Authors’ contribution

B.O and G.F. conceptualized the study; C.G. obtained the yeast isolates; K.A., T.D., and B.O. performed the experiments; B.O., K.A., and G.F. organized and cured the data; writing and original draft preparation, B.O. and G.F.; all the authors contributed editing the manuscript; supervision, project administration, and funding acquisition, G.F. 

## Conflicts of interest

The authors declare that they do not have anything to disclose regarding funding or conflict of interest concerning this manuscript.

## Financial disclosure

No financial interests related to the material of this manuscript have been declared.
